# Diabetes self‐management education and its association with hospital admissions and premature mortality: A scoping review and meta‐analysis

**DOI:** 10.1111/dom.70296

**Published:** 2025-11-24

**Authors:** Gemma A. Lewis, David M. Hughes, Greg Irving, John Wilding, Kevin Hardy

**Affiliations:** ^1^ Department of Cardiovascular and Metabolic Medicine, Institute of Life Course and Medical Sciences University of Liverpool Liverpool UK; ^2^ Department of Diabetes and Endocrinology St Helens Hospital, Mersey and West Lancashire Teaching Hospitals NHS Trust St Helens UK; ^3^ Department of Health Data Science, Institute of Population Health University of Liverpool Liverpool UK; ^4^ Health Research Institute Edge Hill University Ormskirk UK

**Keywords:** diabetes, hospital admissions, mortality, patient education as topic, scoping review

## Abstract

Type 2 diabetes is associated with increased all‐cause hospital admissions and premature mortality. Diabetes self‐management education (DSME) is internationally recommended but its impact on reducing hospital admissions and premature mortality is unclear. This scoping review followed the PRISMA extension for scoping reviews and explores the relationship between DSME, hospital admissions and mortality in adults with type 2 diabetes. Core search terms for type 2 diabetes were combined with terms for DSME, hospital admission, and mortality. Searches were conducted on 10 January 2024, and checked in September 2025, across three electronic databases: CINAHL, MEDLINE, and EMBASE. No restrictions were applied regarding study design, quality, location, or sex. The search yielded 294 records from databases and 66 from manual searches; 42 met inclusion criteria. Evidence regarding hospitalisation was inconsistent, with heterogenous outcome definitions and frequent reporting of admissions as a reason for attrition. Meta‐analysis showed a non‐significant 9% reduction in admission risk among DSME groups. By contrast, meta‐regression found that whether mortality was reported as a primary outcome significantly moderated the treatment effect (*p* = 0.0002), fully accounting for between‐study heterogeneity (τ^2^ = 0; *I* ^2^ = 0%; *R* ^2^ = 100%). A final pooled analysis, restricted to studies with mortality as a primary outcome, demonstrated a ~45% reduction in mortality risk (RR 0.55, 95% CI 0.47–0.63, *p* < 0.0001). DSME may offer a survival benefit for adults with type 2 diabetes, but current evidence does not support an association with reduced hospitalisation. Standardisation of DSME content and outcome reporting is essential to improve comparability.

## 1 INTRODUCTION

Diabetes is a complex condition leading to disability and premature mortality with 7000 excess deaths in the United Kingdom in 2022.^1^ Globally the number of adults living with diabetes has risen sharply over the past 40 years^2^ with the International Diabetes Federation estimating 537 million people are affected worldwide^3^ of whom approximately 4.3 million live in the UK.^4^ Over 90% have type 2 diabetes, with an increased prevalence and multimorbidity risk in areas with high deprivation, poor healthcare access, poorer housing, reduced finances, and lower educational attainment.^2^, ^4^ Individuals with type 2 diabetes are being diagnosed younger and living longer,^2^ likely due to the implementation of secondary prevention guidelines,^5^, ^6^, ^7^, ^8^ but there remains a persistent gap in excess mortality compared to populations without the disease. Diabetes Self‐Management Education (DSME) is internationally recognised as a cornerstone to successful diabetes self‐management, and with pharmacotherapy, fundamental in achieving early glycaemic control and prevention of diabetes‐related micro‐ and macrovascular complications.^7^, ^9^

Landmark studies demonstrate the effectiveness of DSME in improving self‐management skills, reducing diabetes distress and improving health‐related quality of life, with short to medium‐term improvements to glycated haemoglobin (HbA1c).^10^, ^11^, ^12^, ^13^, ^14^ Despite this, there remains limited and conflicting evidence on the longer‐term benefits of DSME, largely due to heterogeneity in programme design, content, duration and delivery. People living with type 2 diabetes frequently present with at least 1 comorbid condition which impacts ability to self‐care,^15^ disease progression, likelihood of hospital admissions and premature mortality.^16^ Whilst a decline in diabetes‐related morbidity and mortality is evidenced in the literature,^2^, ^17^, ^18^ 20% of all UK hospital beds are currently occupied by people with diabetes, a trend replicated globally with hospitalisation rates in those with diabetes 2–6 times higher than other populations.^19^, ^20^ Hospital bed usage is expected to rise to 25% by 2030,^21^ most (92%) are admitted for conditions and illnesses other than diabetes.^18^, ^22^ Similarly, although a global reduction in all‐cause mortality has been observed for both individuals with and without diabetes, an unexplained excess mortality risk persists among people living with diabetes^2^, ^5^, ^6^, ^17^ reflecting the progressive nature of the disease rather than any adverse effect of DSME.

With increasing evidence suggesting primary and secondary management of diabetes is not enough to remove the persistent excess gap in hospital admissions and premature mortality, a comprehensive overview of the long‐term impact of DSME—a fundamental component of effective self‐management^9^—is warranted. Owing to the observational nature of this research question and the lack of standardised global practices in DSME delivery, a scoping review was identified as the most appropriate method to understand the extent of literature available and to identify gaps in knowledge. Therefore, the objective of this scoping review was to systematically map available evidence, key concepts and current knowledge on the relationship between DSME, hospital admissions and premature mortality in adults living with type 2 diabetes.

## 2 METHODS

This scoping review followed the five‐stage process for scoping studies methodological framework^23^ and was developed using the Preferred Reporting Items for Systematic Reviews and Meta‐Analysis Extension for Scoping Reviews^24^ (PRISMA‐ScR) guidelines (protocol available in Data S1). Using a population, intervention, comparator, outcome (PICO) framework as recommended by PRISMA‐ScR, the population comprised adults living with type 2 diabetes; the intervention was structured DSME; the comparator was attendance at DSME versus no DSME or partial completion; and the outcomes were hospital admissions or mortality.

### 2.1 Research question

What is the extent of literature that exists about the association between DSME attendance, hospital admissions and premature mortality in people living with type 2 diabetes?

### 2.2 Information sources and searches

To identify potentially relevant evidence the following bibliographic databases were searched: CINAHL, MEDLINE and EMBASE. Search strategies were drafted and refined with support from an experienced librarian before being conducted on 10 January 2024 and checked in September 2025. Core search terms for type 2 diabetes were combined using the Boolean operator AND with terms relevant to diabetes self‐management education AND mortality or hospital admissions.

We identified primary studies and grey literature relevant to DSME for type 2 diabetes. Systematic reviews were initially included and used to confirm that papers potentially meeting the inclusion criteria had not been missed by our search. Final reference lists were exported into Rayyan software^25^ and duplicates (*n* = 30) removed. Reference lists of final documents were reviewed to identify other forms of literature. Final search strategies for CINAHL PLUS and MEDLINE can be found in Data S1. Since MEDLINE's indexed content overlaps with PubMed, and CINAHL and EMBASE ensure broad coverage of clinical and allied health literature, PubMed and Web of Science were not searched to avoid redundancy.

### 2.3 Literature selection

A two‐stage process was adopted for the screening of potentially relevant documents. To ensure consistency throughout the screening process, two reviewers (Gemma A. Lewis and Kevin Hardy) reviewed the same 20 publications, discussed the results, and refined the screening strategy prior to the screening of titles and abstracts.

During the 1st stage, the title and abstract of all eligible articles were screened using Rayyan software.^25^ Blinding was enabled in Rayyan so that each reviewer's inclusion and exclusion criteria were concealed from the other during screening ensuring an independent assessment. Documents were only excluded at this stage if they did not pertain to DSME. If a title appeared relevant, but no abstract was available, this article was advanced to the second stage. Any conflicts were discussed, amended and where agreement could not be reached a third reviewer was available, although this was not required during this scoping review.

During the second stage two reviewers (GL and KH) independently and blindly screened all full‐text articles against the inclusion and exclusion criteria using Rayyan software. Authors of seven papers were contacted for further information. Agreement between the two reviewers was calculated using Kappa statistics with a Cohen Kappa score of 0.81.

### 2.4 Inclusion and exclusion criteria

Adults aged 18 years or older were included where they had a confirmed diagnosis of type 2 diabetes. Studies focusing on diabetes prevention education were excluded. We considered all study designs about DSME globally where outcomes for type 2 diabetes were reported independently of other conditions or types of diabetes. It is however recognised that prevalence, risks and approaches to supported self‐management differ widely.

Our review was not specific to gender, sex or ethnicity and studies published from 1 January 2004 onwards were included; but we recognize that DSME delivery may have changed over the last 20 years. Studies were included where they were written in English or where an English transcription was available. Where multiple papers were available reporting findings from the same study, the most recent was included, for example, 5‐year follow up vs. 2‐year follow up; the 5‐year follow up was accepted.

DSME is the term recognised for behavioural interventions to support patients' self‐management skills in type 2 diabetes, but there is no nationally or internationally agreed content. To be classified as DSME for this review the study had to indicate that the programme was evidence‐based, structured (e.g., approved curriculum with stated learning outcomes and objectives), and delivered by trained educators and fundamentally different from patient advice given during a routine consultation. Programmes did not need to be accredited.

Studies were excluded where the focus was on carbohydrate counting, type 1 diabetes, or where the focus was comparing different methods of delivering education rather than the outcome of DSME education. In cases where study designs included comparisons between control, DSME, and DSME combined with medication or additional interventions, only the data comparing control versus DSME were extracted during the review process.

### 2.5 Data charting process

A data charting table was developed by the first reviewer (GL) prior to being discussed, checked and piloted by two reviewers (GL and KH) on a random sample of 10 articles and modified as required based on feedback. Full data abstraction began only after agreement had been reached. GL conducted data extraction with KH providing verification of included papers, discussing results in an iterative process aligning with the review focus. Studies were grouped by outcome of interest and summarised by setting, study design, outcome measures, admissions and mortality details and author conclusions. Details of the included studies are summarised by outcome: Table 1 hospital admissions; Supporting information Table SI mortality. Meta‐analysis for hospital admissions and mortality outcomes was conducted using the reported admissions and mortality count data reported in intervention and control groups. To address variability in follow‐up duration, meta‐regression was performed on mortality data including mean follow‐up time and study outcomes (mortality as a primary endpoint versus reason for attrition) as covariates.

**TABLE 1:** Extracted data for hospital admissions.

Author & year	Country	Aims	Sample description	Outcome measures	Admissions	Author conclusions/recommendations	DSME characteristics
Adepoju,^41^ 2014	USA	Does DSME delay occurrence of acute events necessitating hospitalisation	376 adults Mean age 57 55% female	Time to 1st hospitalisation	Intervention significantly (*p* = 0.002) reduced the odds of hospitalisation (hazard ratio: 0.10) and prolonged time to admission.	Higher HbA1c, race, comorbidities and increasing age linked to shorter time to admission. CDSMP is likely to be effective within a short time span (2 years) of prolonging time to hospitalisation in T2DM.	Chronic disease self‐management programme (CDSMP) Face to face, group format Delivered by a disease management coach 6 sessions totalling 15 h Measurement of completion not stated. ITT analysis used
Colungo,^29^ 2018	Spain	Effectiveness of therapeutic DSME in newly diagnosed type 2 diabetes	161 adults Mean age 65 45% female	HbA1c, weight, physical activity levels	Hospital admissions in intervention group 0%, control group 4.5%	PAET‐Debut DM2 is effective in improving clinical factors and improving satisfaction	PAET‐Debut DM2 Face to face, group Delivered by GP and nurse 6 sessions totalling 12 h Completion based on 100% attendance
El Toony,^39^ 2018	Egypt	Effectiveness of pre‐Ramadan DSME on acute complications including risk of hypoglycaemia	320 adults Mean age 50.1 75% female	Impact of fasting on biological parameters / hypo	Hospital admissions intervention group 0/120 vs. control group 4/200	Pre‐fasting education has a positive impact on patients. Patients require support to fast safely	Programme name not stated Face to face, 1:1 format Facilitator background not stated 1 session lasting 30 min Measurement of completion not stated
Fu,^43^ 2015	China	Effectiveness of DSME on knowledge and self‐management	39 adults Mean age 70 65.5% female	Diabetes knowledge and self‐management	Dropouts included 2 hospital admissions	DSME had positive effects on Chinese population with type 2 diabetes. Should be incorporated into routine clinical care	Programme name not stated (content guided by China Guideline for Diabetes Care and Education) Face to face, group format Delivered by nurses 6 sessions totalling 6 h Completion based on a minimum 60% attendance
Gamboa Moreno,^44^ 2019	Spain	Efficacy of the Spanish DSME versus usual care in type 2 diabetes	157 adults Mean age 64 40% female	HbA1c, cardiovascular risk factors; medication use; quality of life; self‐efficacy; physical activity levels; GP appointments; hospitalisations)	No meaningful difference (*p* = 0.787) in hospital admissions at 12 and 24 months between intervention and control groups	HbA1c reductions are difficult to obtain in adequately controlled patients. HbA1c as outcome may be more suitable for studying poorly controlled groups.	Spanish version, Chronic disease self‐management programme (CDSMP) Face to face format, not stated if group or 1:1 Delivered by healthcare professional and peer with lived experience of diabetes 6 sessions totalling 6 h 80% attended a minimum of 4 sessions. ITT analysis used
Goudswaard,^34^ 2004	Netherlands	Efficacy of a 6‐month DSME in type 2 diabetes	58 adults Mean age 65.3 66% female	HbA1c	Hospital admission reported as reason for drop out (intervention group 0/28, control group 1/30)	Education effective in reducing HbA1c and delaying insulin therapy for patients on maximum oral therapy, however effect reduced at 1 year. Short term education requires regular reinforcements.	Programme name not stated (developed in collaboration with Dutch Foundation of Diabetes Nurses) Face to face, 1:1 format Delivered by diabetes nurse 6 sessions totalling 2.5 h Completion based on 100% attendance
Guo,^30^ 2014	China	Efficacy of DSME in insulin treated type 2 diabetes	1511 adults Mean age 57.2 49% female	HbA1c	Hospital admissions included as a reason for dropout (intervention group 3/731, control group 7/726)	DSME can improve self‐management, glycaemic control and medication compliance	The OPENING program Face to face, 1:1 format Delivered by nurses 7 sessions, total length not stated Patients included in analysis where they had attended at least one session. ITT analysis used
Hamid, 2014	Samoa	Impact on diabetes control and healthcare usage 1 year post DSME	268 adults Mean age 55 67% female	Primary care physician visits, emergency department attendance and hospitalisation	Change in hospital admissions not significant (*p* = 0.7) (intervention group 11/104, control group 15/164)	DSME significantly increased primary care visits and decreased emergency department visits among those with high emergency department usage the previous year.	Programme name not stated (modelled on National Diabetes Education Program) Face to face, 1:1 format Delivered by a community health worker Number of sessions and programme length not stated Measurement of completion not stated. ITT analysis used
Johansson,^51^ 2017	Austria	Is peer support in addition to DSME effective in reducing medication use, admission and length of stay	337 adults Mean age not stated % female not stated	Cost analysis of previous trial	Total number of hospital admissions intervention group 174 (mean 1.5), 229 (1.6) in control group *p* = 0.391. Length of hospital stay *p* = 0.041 intervention group 65.3 (41.8) to control group 105.4 (69.1)	Group education with peer support can reduce all cause hospitalisations (not significantly) with a significant reduction in length of stay. This constitutes a cost saving.	Aktivtreff Diabetes Face to face, group format Delivered by peer supporters and healthcare professionals Number of sessions and programme length not stated Measurement of completion not stated.
Kellow,^52^ 2020	Australia	Evaluation of culturally tailored DSME	34 adults Mean age 69 65% female	HbA1c, diabetes distress and self‐related health status	Dropouts included 1 hospital admission	Cultural learning orientations and community expectations should be incorporated into diabetes interventions.	Not Scared of Sugar Face to face format, not stated if group or 1:1 Delivered by healthcare professional and diabetes educator 5 sessions totalling 10 h Measurement of completion not stated. ITT analysis used
Lalic,^26^ 2017	Serbia	Effect of self‐monitoring of blood glucose and intensive DSME on hospitalisations and cardiovascular risk factors	289 adults Mean age 56.7% female not stated	HbA1c, weekly frequency of SMBG, hospitalisations, waist circumference, blood pressure, lipid levels, albumin secretion and diabetes distress.	Number of admissions due to diabetes complications significantly decreased (0.28 ± 0.06 vs. 0.11 ± 0.03, *p* < 0.05). Rate of admissions significantly reduced at 6 months (0.46 ± 0.07 vs. 0.10 ± 0.03, *p* < 0.01).	Education and SMBG together significantly reduced HbA1c, increased SMBG frequency improved quality of life and reduced the number of hospital admissions.	Programme name not stated (10‐day programme) Face to face format, not stated if group or 1:1 Facilitator background not stated 10 sessions, programme length not stated Measurement of completion not stated.
Lorig,^45^ 2009	USA	Effectiveness of community based DSME	345 adults Mean age 66.7 66% female	Health status (HbA1c, weight, depression, fatigue, hypo), Self‐management behaviours and self‐efficacy	No significant relationship between DSME attendance and days in hospital at 6 months (intervention group 0.00 (4.31) vs. control group −0.143 (1.75) *p* = 0.152), or 12 months *p* = 0.492	DSME intervention demonstrated sustained improvements at 12 months in depression scores, communication with physician, healthy eating and self‐efficacy. There were no significant differences in healthcare utilisation.	Spanish version, Chronic disease self‐management programme (DSMP) Face to face format, not stated if group or 1:1 Delivered by peer supporters 6 sessions totalling 18 h Measurement of completion not stated. ITT analysis used. Mean of 4.9 sessions attended
Magee,^50^ 2011	USA	Feasibility of DSME on emergency department visits, knowledge and HbA1c	360 adults Mean age 59.9 82.1% female	Retention of participants, guideline targets for HbA1c, BP and lipid levels, medication adherence, self‐efficacy scores and emergency department visits	Hospitalisations pre‐post intervention reduced but not significant *n* = 13 (5.1%) vs. post intervention *n* = 8 (3.1%) *p* = 0.25.	Significant reductions in HbA1c and a two‐thirds reduction in emergency department visits. Community location like libraries offer accessible location for DSME delivery.	Concise ABCs of diabetes DSME program Face to face, group format Delivered by diabetes educators 2 sessions totalling 5 h Completion based on 100% attendance
McGowan,^46^ 2015	Canada	Effectiveness of peer DSME on self‐efficacy and behaviours	252 adults Mean age 64.2 61.8% female	HbA1c	Post hoc analysis of admissions and nights in hospital showed marginal interaction with group however did not reach significance.	Fatigue, cognitive symptoms management, self‐efficacy, communication with physicians and empowerment improved significantly.	Stanford Diabetes self‐management programme Face to face, group format Facilitator background not stated 6 sessions totalling 12 h Measurement of completion not stated
Timple,^53^ 2022	USA	Identification of 30‐day readmission predictors in type 2 diabetes	400 adults Mean age 78.6 49% female	Hospital re‐admission	DSME decreased likelihood of 30‐day re‐admission by 2.429 times (95% CI, 1.046–5.639) with a significant *p* = 0.006 with a weak association between DSME and readmission.	Patients who attend DSME are less likely to be readmitted to hospital. DSME should be offered on a continual basis whilst both in hospital and in community to reduce re‐admission rates, emphasising need for	Programme name not stated Face to face, 1:1 format Delivered by peer supporters Number of sessions and programme length not stated Measurement of completion not stated
Wong,^48^ 2016	Hong Kong	Is DSME associated with a lower all cause admission in type 2 diabetes	12 125 adults Mean age 63.9 56.8% female	Hospitalisation rate	During a 30.5 month follow up intervention group had a lower incidence of initial hospitalisation event (22.1 vs. 25.2%; hazard ratio 0.879; *p* < 0.001) and fewer hospitalisation episodes (incidence rate ratio 0.854; *p* < 0.001); 16.9 hospitalisations per 100 in intervention vs. 20.0 in control groups.	DSME effective at delaying time to first hospitalisation and in reducing frequency of admissions and reduction in associated direct medical costs in a real‐world primary care setting.	Patient empowerment programme (PEP) Face to face, group format Delivered by nurse and community experts 6–8 sessions totalling 15 h Patients must have completed at least 1 session to be included in analysis, ITT analysis used

Abbreviation: ITT, intention to treat.

### 2.6 Critical appraisal

The mixed methods appraisal tool (MMAT) was adopted to assess the methodological quality of included papers allowing for a unified appraisal. As per recommendations two reviewers independently judged paper quality against the MMAT screening questions, before commencing the tailored questions for randomised control trials (RCTs), non‐randomised studies or quantitative descriptive studies (Table 2).

**TABLE 2:** Mixed methods appraisal table (MMAT).

	Screening questions		Randomised controlled trials					Non‐randomised studies					Quantitative descriptive studies				
	Are there clear research questions?	Do the collected data allow to address the research questions?	Is randomisation appropriately performed?	Are the groups comparable at baseline?	Are there complete outcome data?	Are outcome assessors blinded to the intervention provided?	Did the participants adhere to the assigned intervention?	Are the participants representative of the target population?	Are measurements appropriate regarding both the outcome and intervention (or exposure)?	Are there complete outcome data?	Are the confounders accounted for in the design and analysis?	During the study period, is the intervention administered (or exposure occurred) as intended?	Is the sampling strategy relevant to address the research question?	Is the sample representative of the target population?	Are the measurements appropriate?	Is the risk of nonresponse bias low?	Is the statistical analysis appropriate to answer the research question?
Adepoju, 2014^41^	Y	Y											Y	N	Y	Y	Y
Adolfsson, 2006^28^	Y	Y	Y	Y	Y	Y	Y										
Blackberry, 2013^38^	Y	Y	Y	Y	Y	N	N										
Chao, 2014^54^	Y	Y	CT	N	Y	N	CT										
Clancy, 2007^32^	Y	Y	Y	CT	Y	N	CT										
Clark, 2004^55^	Y	Y	CT	Y	Y	N	CT										
Colungo, 2018^29^	N	CT						N	CT	N	Y	Y					
Cooper, 2008^56^	Y	Y	CT	CT	N	N	N										
Crasto, 2011^40^	Y	Y			Y	Y	N										
Crowley, 2013^57^	Y	Y	Y	Y	Y	N	N										
Deakin, 2006^42^	Y	Y	Y	Y	N	Y	N										
Edelman, 2015^58^	Y	Y	CT	Y	N	N	N										
El Toony, 2018^39^	N	CT						N	Y	CT	Y	Y					
Fu, 2015^43^	Y	Y						N	Y	N	Y	Y					
Gagliardino, 2013^33^	Y	Y	CT	N	N	N	CT										
Gamboa Moreno, 2019^44^	Y	Y	Y	Y	Y	N	N										
Gehlawat, 2019^59^	Y	Y	Y	N	Y	N	Y										
Goudswaard, 2004^34^	Y	Y	Y	N	Y	N	Y										
Guo, 2014^30^	Y	Y	Y	Y	Y	N	CT										
Hamid, 2014^60^	Y	Y	CT	Y	Y	N	CT										
Jaipakdee, 2015^35^	Y	Y	CT	N	Y	N	Y										
Johansson, 2017^51^	Y	Y											Y	N	Y	Y	Y
Kellow, 2020^52^	Y	Y						N	Y	N	CT	Y					
Khunti, 2012^13^	Y	Y						Y	Y	N	Y	Y					
Lalic, 2017^26^	Y	Y											N	N	Y	Y	Y
Lorig, 2009^45^	Y	Y	CT	Y	N	CT	N										
Magee, 2011^50^	Y	Y						N	Y	N	Y	Y					
McGowan, 2015^46^	Y	Y	Y	Y	N	N	Y										
Pearson, 2021^27^	Y	Y	Y	Y	Y	N	Y										
Perez‐Escamilla, 2015^36^	Y	Y	Y	Y	N	N	N										
Perman, 2011^61^	Y	Y											Y	N	Y	Y	Y
Prezio, 2014^62^	Y	Y											Y	N	Y	Y	Y
Rygg, 2012^63^	Y	Y	Y	N	Y	N	N										
Samuel‐Hodge, 2009^64^	Y	Y	Y	Y	Y	N	N										
Sarkadi, 2004^65^	Y	Y	N	N	Y	N	CT										
Sperl‐Hillen, 2013^37^	Y	Y	CT	Y	Y	N	N										
Timple, 2022^53^	Y	Y											Y	Y	Y	Y	Y
Trento, 2004^66^	N	CT	CT	N	N	CT	Y										
Wong, 2016^48^	Y	Y											Y	Y	Y	Y	Y
Wong, 2016^31^	Y	Y											Y	Y	Y	Y	Y
Wong, 2015^47^	Y	Y											Y	Y	Y	Y	Y

*Note*: MMAT rating: Y, yes ‐ shaded green; N, no ‐ shaded red; CT, Can't tell ‐ shaded yellow.

## 3 RESULTS

### 3.1 Evidence characteristics

**FIGURE 1:**
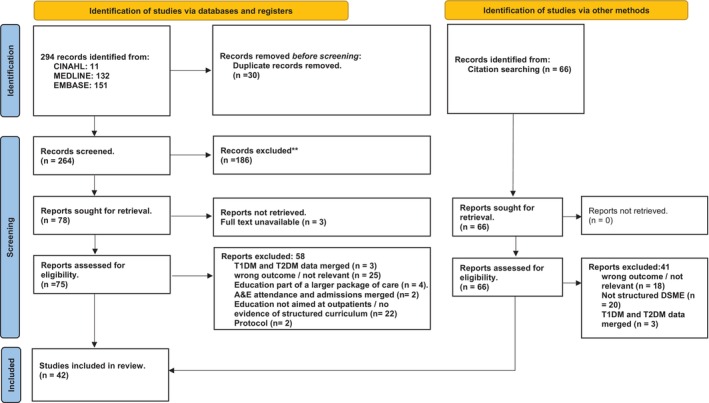
PRISMA flow diagram.^24^

A systematic search of three databases identified 276 records, with an additional 66 records retrieved through hand‐searching key journals, reference lists and diabetes‐related websites. Forty‐two publications met the final inclusion criteria (Figure 1). Most studies originated from high‐income countries (63%), with the United States (*n* = 10) and the United Kingdom (*n* = 6) contributing the greatest number of studies (Data S1). Two RCTs from India and Samoa, and one non‐randomised study from Egypt, represented the only contributions from lower‐middle‐income countries. Overall, the review included 26 RCTs, six non‐randomised studies, and nine quantitative descriptive studies. Among these, 25 RCTs, four non‐randomised controlled trials, and seven quantitative descriptive studies reported receiving research funding.

The earliest publication included is by Trento et al. in 2004 and the most recent by Timple et al. in 2022. Publication numbers peaked in 2015 and have plateaued over the last 5 years.

Of the 42 included studies, all but two specifically focused on individuals living with type 2 diabetes. A 6‐month observational study by Lalic et al.^26^ included participants with both type 1 (*n* = 57) and type 2 diabetes (*n* = 289), while a single‐centre study by Pearson et al^27^ involved 69 participants with type 1 diabetes and 60 with type 2 diabetes. In both studies, outcomes for individuals with type 1 and type 2 diabetes were reported separately, enabling their inclusion in this scoping review.

All included studies were peer‐reviewed and primarily employed purposeful sampling (*n* = 22) or consecutive sampling (*n* = 13). Whilst all studies included both male and female participants, females represented the majority of the overall sample (57.3%), with a mean participant age of 60.1 years.

Diabetes duration was reported as a baseline characteristic in 32 studies, with a mean duration of 7.4 years. There was substantial heterogeneity in inclusion criteria, ranging from individuals who were newly diagnosed,^13^, ^28^, ^29^ insulin therapy,^26^, ^30^ experiencing severe hypoglycaemia,^27^ or living with obesity.^31^ The most frequently reported (*n* = 7) inclusion criterion was a HbA1c level >7%.^32^, ^33^, ^34^, ^35^, ^36^, ^37^, ^38^

### 3.2 Structured education

Considerable heterogeneity was observed within DSME programme design, delivery format, facilitator background, duration, and benchmark of educational completion (Table 2). Whilst most DSME programmes were delivered face to face, in group settings, several incorporated individual or telephone components. One study used a hybrid approach combining in‐person and remote delivery.^38^ DSME was most frequently delivered by nurses, members of the MDT with a handful of studies opting for co‐delivery models with diabetes educators and peer support workers with lived experience of diabetes. Community health workers were often utilised in culturally tailored or community‐based programmes to support engagement and delivery. Variability in programme length ranged from a single session^13^, ^27^, ^39^, ^40^ to 17 sessions,^36^ with total duration ranging from 30 min^39^ to over 24 h^32^ making direct comparisons challenging. A large proportion of studies offered 6 sessions^29^, ^31^, ^34^, ^35^, ^41^, ^42^, ^43^, ^44^, ^45^, ^46^, ^47^, ^48^ as part of their structured curriculum; however overall session duration and total contact time were poorly reported within the included papers.

Whilst no standardised benchmark currently exists for measures of completion for DSME programmes,^49^ definitions of individual programme completion varied widely, ranging from at least one session^31^, ^47^, ^48^ to 100% attendance^29^, ^34^, ^50^ with most studies (72%) not explicitly reporting adherence thresholds and the majority adopting intention to treat (ITT) analyses, including those with low participant adherence or incomplete reporting of session completion. While ITT preserves the benefits of randomisation and minimises bias, its use in DSME with poor intervention adherence may attenuate the observed effects and dilute the estimated impact of DSME on hospital admissions and mortality outcomes. Consequently, the true effectiveness of DSME may be underestimated in these studies, complicating the interpretation of the results.

### 3.3 Critical appraisal within sources of evidence

Many studies (*n* = 13) listed both biological and patient‐reported outcomes as their primary and secondary endpoints. HbA1c was the most studied outcome, assessed in 26 studies, despite previous evidence suggesting DSME's limited effectiveness on HbA1c—though this is beyond the scope of this review. Seven studies also evaluated changes to weight, BMI and physical activity levels.^13^, ^29^, ^33^, ^35^, ^38^, ^42^, ^66^ Patient‐reported outcome measures were the second most frequently used (*n* = 15) encompassing quality of life,^35^, ^44^, ^66^ diabetes knowledge and self‐management skills,^28^, ^43^, ^44^, ^45^, ^46^, ^55^, ^56^, ^59^, ^64^, ^66^ medication adherence,^50^, ^57^ patient activation^63^ and diabetes distress.^26^, ^37^

### 3.4 Hospital admissions

Of the 16 studies presented in Table 1, only six explicitly included hospital admissions as a primary or secondary outcome measure. These studies varied widely in how they defined and measured hospital admissions, including time to first hospitalisation,^41^ number or rate of hospital admissions,^26^, ^44^, ^48^, ^60^ and hospital readmission rates.^53^

Higher HbA1c, age, and comorbidities were linked to shorter time to first hospitalisation, indicating that DSME might help delay initial hospital admissions in type 2 diabetes.^41^ A large primary care cohort study found DSME to be effective in delaying hospitalisations and reducing admission frequency, alongside lowering direct medical costs.^48^ Additionally combined self‐monitoring of blood glucose and education was shown to reduce the number of hospital admissions, in addition to improving clinical outcomes such as HbA1c and quality of life.^26^ Furthermore, patients attending DSME were less likely to be readmitted within 30 days, supporting the value of continuous education to reduce hospital readmissions.^53^

The remaining 10 studies mentioned hospitalisation only as a reason for participant dropout, without detailed analysis or reporting of admission outcomes. This inconsistency in how hospital admissions are measured and reported limits the ability to draw robust conclusions on the impact of DSME on hospitalisation rates. However, the fact that hospitalisation contributed to dropout highlights it as a potential barrier to sustained engagement in DSME programs. This suggests that patients with more severe or unstable diabetes may struggle to participate fully, which could introduce bias if not properly accounted for. These findings underscore the need for future research to systematically capture and analyse hospitalisation events to better understand their impact on DSME effectiveness and patient retention.

Lack of a standardised definition and measurement approach meant only five studies^30^, ^34^, ^39^, ^51^, ^60^ were included in the meta‐analysis limiting the synthesis of hospital admissions. A random effects model compared admission rates between intervention and control groups across five studies. The pooled estimate demonstrated a ~ 9% reduction in hospital admission risk in the intervention group compared to the control (RR 0.91, 95% CI 0.76–1.10, *p* = 0.34). This difference was not statistically significant. (Hospital admissions forest plot in Data S1).

### 3.5 Mortality

Table SI summarises 29 studies reporting mortality data, with five designating mortality as a primary or secondary outcome measure. Outcomes included all‐cause mortality, cardiovascular mortality, and long‐term mortality projections DSME attendance was associated with a 33% reduction in crude all‐cause mortality over 6 years in participants completing ≥75% of the program.^61^ Simulated models found DSME to be cost‐effective over 20 years, though mortality reductions were not statistically significant.^62^

Real‐world cohort studies, including Pearson et al. (2021), reported significant reductions in cardiovascular and all‐cause mortality following DSME participation, particularly among patients with severe hypoglycaemia or cardiovascular disease.^27^ Notably, a 2016 Hong Kong study found a 41% lower all‐cause mortality risk in patients attending at least 1 DSME session (HR 0.589, 95% CI 0.389–0.915),^31^ consistent with their earlier six‐year follow‐up which suggested a 43% reduction (HR 0.564, 95% CI 0.445–0.715).^47^ Similarly, an Argentinian study reported mortality rates of 33.3% in DSME groups vs. 51% in controls over 6 years; however, this effect was reduced to 18% following adjustment for covariates (HR 0.82, 95% CI 0.61;1.08).^61^ Several studies observed attenuated effects after multivariate adjustment, highlighting the potential influence of baseline health status and socioeconomic factors as confounders.

A pooled analysis of 29 studies including 43 516 participants identified a total of 607 deaths. Twenty‐eight studies were initially included in a random‐effects meta‐analysis, as one study was excluded because it reported simulated mortality data rather than observed outcomes. The analysis demonstrated that DSME intervention was associated with a 27.6% relative reduction in mortality (RR 0.74, 95% CI 0.56–0.99, *p* < 0.05), with a reasonably consistent direction of effect in favour of DSME. Although between‐study heterogeneity was low to moderate (τ^2^ = 0.1655; *I* ^2^ = 27.6%), meta‐regression was conducted using follow‐up duration as a moderator. The association between follow‐up time and log risk ratio was not statistically significant (*p* = 0.56), indicating no meaningful change in effect size with increasing follow‐up duration. Residual heterogeneity remained moderate (τ^2^ = 0.1923; *I* ^2^ = 46.3%), and the model explained 0% of the between‐study variability (*R* ^2^ = 0%).

**FIGURE 2:**
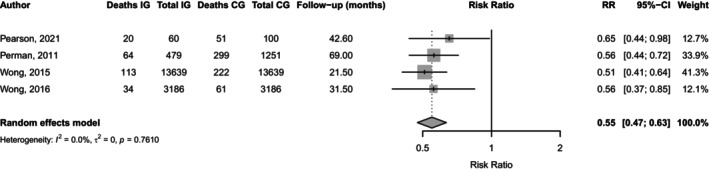
Meta‐analysis of pooled mortality in primary outcome studies.

By contrast, study outcome (mortality as a primary outcome vs. incidental reporting) significantly moderated the treatment effect (*p* = 0.0002). Studies which listed mortality as a primary outcome (*n* = 4) showed a 49% reduction in risk (RR = 0.51; 95% CI: 0.36, 0.73), whereas studies that reported mortality incidentally (e.g., as a reason for dropout) (*n* = 24) showed no significant effect. Inclusion of this moderator fully accounted for between‐study heterogeneity (τ^2^ = 0; *I* ^2^ = 0%; *R* ^2^ = 100%). Based on this finding, the final analysis, restricted to studies with mortality as a primary outcome, demonstrated a significant ~45% reduction in mortality risk (RR 0.55, 95% CI 0.47–0.63, *p* < 0.0001), as illustrated in Figure 2.

### 3.6 Critical appraisal

Reviewers (Gemma A. Lewis and Kevin Hardy) critically appraised the included papers and whilst the overall methodological quality of included studies was moderate to high, particularly among RCTs, there is variability regarding the completeness of outcome data, confounding variables, and design details. Thirty‐nine of the included papers passed the initial screening questions confirming they had clearly defined research questions and collected data that addressed those questions. The remaining three studies^29^, ^39^, ^66^ did not explicitly state a research question, resulting in a “No” response to the first screening question and a corresponding “Cannot tell” for the adequacy of collected data.

Among the RCTs, most reported appropriate randomisation and comparable baseline groups; however, only three stated whether outcome assessors were blinded,^28^, ^40^, ^42^ and participant adherence was often underreported, which impacted methodological rigour.

Only one non‐randomised study included a sample representative of the target population,^13^ limiting generalisability. None of the non‐randomised studies reported complete outcome data, suggesting potential bias and affecting the validity of their findings. Despite this, five of six papers included appropriate measures to evaluate the intervention, accounted for confounders and ensured the intervention was delivered as intended, indicating high intervention fidelity.

Most quantitative descriptive studies had appropriate sampling strategies and used reliable measurements for primary outcomes, often HbA1c. However, hospital admissions and premature mortality were frequently reported post hoc or as reasons for dropout limiting the usefulness of this appraisal question. All nine papers conducted appropriate statistical analysis supporting the research question.

## 4 DISCUSSION

The persistent disparity in the rate of hospital admissions and premature mortality among people living with type 2 diabetes is well documented.^2^, ^17^, ^18^ Existing literature provides evidence that DSME improves quality of life, self‐management skills, and short‐term HbA1c, but persuasive evidence of its impact on hospital admissions and premature mortality remains uncertain. This review sought to address this gap by mapping the available literature on the relationship between DSME participation, hospitalisation, and mortality outcomes.

The overarching aim of DSME is to empower individuals with type 2 diabetes to adopt sustainable lifestyle changes that prevent or delay complications. Although numerous studies have examined the effectiveness of individual DSME programmes, these largely focus on behavioural and clinical endpoints such as HbA1c.^14^, ^42^, ^67^ Sustained improvements in HbA1c are associated with long‐term reductions in microvascular and macrovascular complications,^68^ so it is plausible that DSME could also reduce hospital admissions and premature mortality, but current evidence remains inconclusive.

Findings on hospitalisation were inconsistent, complicated by varied outcome definitions and by frequent reporting of hospitalisation as a reason for dropout rather than a measured endpoint. The omission of hospitalisation as a defined endpoint may have introduced bias, as participants experiencing hospital admission were systematically excluded from follow‐up, potentially underestimating the true frequency of hospitalisation events. DSME interventions were heterogeneous and lacked a clear, standardised definition, and the methodological design and quality of studies varied considerably. Meta‐analysis confirmed current evidence suggests that DSME is not associated with a meaningful reduction in hospital admissions. This represents a key shortcoming in the existing literature on DSME's impact on hospitalisation and is consistent with prior reports, including those from the Joint British Diabetes Societies for Inpatient Care, which concluded that DSME has limited effectiveness in reducing the high hospitalisation burden associated with type 2 diabetes.^16^

By contrast, the pooled analysis demonstrated a highly significant 45% relative reduction in mortality among DSME participants. Importantly, this estimate excluded simulated mortality data strengthening the robustness of the finding. To reduce residual heterogeneity, studies that reported mortality incidentally (e.g., as a reason for drop out) were excluded from the pooled analysis. Whilst this result is encouraging the evidence was based on a small number of large cohort studies from high‐income countries, limiting generalisability to low‐income settings where diabetes prevalence is rising rapidly. These findings while promising should be interpreted with caution. Nonetheless, supportive evidence from large real‐world cohorts in Asia and Latin America suggests a potential survival benefit, although effect sizes reduced in some studies following adjustment for covariates.^31^, ^47^, ^61^

The observed reduction in mortality among DSME participants may be mediated through improvements in self‐management behaviours, medication adherence, and self‐efficacy, which collectively support better control of microvascular and macrovascular complications.^47^, ^69^ These benefits may result from improved metabolic control and empowerment in self‐care, enabling individuals to recognise and respond to early signs of deterioration.^47^ In contrast, hospital admissions among people with diabetes are often driven by a broader set of acute events, infections and comorbid conditions not directly related to diabetes itself and therefore less likely to be influenced by DSME participation.^18^ Consequently, DSME may exert its greatest impact through sustained behavioural and clinical improvements that reduce long‐term mortality risk rather than short‐term hospitalisation rates.

Of the 42 studies included in this review, 16 assessed hospital admissions and 28 assessed mortality. However, the lack of standardisation in DSME—both nationally and internationally—remains a persistent challenge. DSME varies widely in terms of content, delivery methods, facilitator training, and reporting standards, with no nationally or internationally mandated curriculum or accreditation, further limiting comparability and replication of outcomes across studies. This variability across studies complicates pooled analyses. Differences in programme design, facilitator training, and definitions of programme completion likely influence outcomes and may contribute to the inconsistent findings observed for both hospitalisation and mortality. These limitations have been highlighted in previous systematic reviews, which similarly identified heterogeneity as a key barrier to drawing firm conclusions.^67^, ^69^, ^70^, ^71^

Compared with earlier reviews, this review draws upon a larger pooled sample and includes a high proportion of RCTs. Nevertheless, variability in methodological quality warrants cautious interpretation of the findings. Furthermore, most included studies were conducted in high‐income countries, limiting generalisability to low‐ and middle‐income settings where the burden of diabetes is rapidly increasing. Addressing this evidence gap will require robust longitudinal observational studies and pragmatic trials in real‐world settings, with hospitalisation and mortality pre‐specified as primary endpoints.

This review uniquely collates DSME characteristics and captures measures of programme completion – an area not typically addressed in previous reviews. While most studies (*n* = 23) adopt ITT analysis, which, while methodologically vigorous may underestimate the true effect of DSME on hospital admissions and mortality due to variable levels of participant engagement.

Overall, this scoping review identifies key gaps in the evidence base, particularly the lack of robust data linking DSME to reductions in hospital admissions and mortality. These findings highlight the need for standardisation of DSME content, delivery and reporting to improve comparability across studies and enable more meaningful interpretation of outcomes.

Although published standards for DSME exist,^72^ these frameworks are not consistently adopted or mandated across healthcare systems, contributing to variability in programme design, delivery, and evaluation. Policymakers could use these findings to implement standardised DSME frameworks by providers, facilitating consistent measurement of programme completion, participant engagement, and clinical effectiveness, and supporting integration into national reporting systems such as the National Diabetes Audit. While the NDA and Quality and Outcomes Framework (QOF) already capture key indicators, linking DSME to standardised monitoring could further enhance benchmarking, accountability, and quality improvement.

Integrating DSME into broader healthcare strategies remains essential to maximize benefits, while recognizing that hospital admissions may be influenced by factors beyond DSME alone. To our knowledge, this is the first review to collate evidence on the association between DSME participation, hospitalization, and mortality outcomes in the general adult population with type 2 diabetes. These findings provide a foundation for guiding future research priorities, shaping policy development, and promoting equitable implementation of DSME programs across diverse healthcare settings.

## FUNDING INFORMATION

No funding was received for this study.

## CONFLICT OF INTEREST STATEMENT

Greg Irving is the National NIHR Research Delivery Network lead for General Practice. John P. H. Wilding reports consultancy/advisory board work for the pharmaceutical industry contracted via the University of Liverpool in the last 36 months (no personal payment) for Alnylam, Amgen, AstraZeneca, Boehringer Ingelheim, Cytoki, Kailera, Lilly, Menarini, Metsera, Napp, Novo Nordisk, Pfizer, Prosciento, Response Pharmaceuticals, Rhythm Pharmaceuticals, Saniona, Shionogi and Ysopia; funding for clinical trials from Amgen, AstraZeneca and Novo Nordisk; and personal honoraria/lecture fees from AstraZeneca, Boehringer Ingelheim, Medscape, Novo Nordisk and Menarini. John P. H. Wilding is past president of the World Obesity Federation, a member of the Association for the Study of Obesity, Diabetes UK, EASD, ADA, Society for Endocrinology and the Rank Prize Funds Nutrition Committee. From 2009 to 2024 he was national lead for the Metabolic and Endocrine Specialty Group of the UK NIHR Clinical Research Network. Gemma A. Lewis, David M. Hughes and Kevin Hardy report no conflicts of interest.

## Supporting information


**DATA S1.** Supporting Information.

## Data Availability

All data supporting the findings of this scoping review are available within the article and its supplementary materials. No primary data were generated or analysed in this study.
