# Supplementary material for: Diabetes self‐management education and its association with hospital admissions and premature mortality: A scoping review and meta‐analysis
Source: Diabetes Obes Metab. 2025 Nov 24;28(2):850–64. doi: 10.1111/dom.70296 (PMC12803649; doi:10.1111/dom.70296)
Supplement: Supplementary file 1 — DATA S1. Supporting Information. [file DOM-28-850-s001.zip › Medline search strategy.docx]

MEDLINE search strategy

| [**Search ID#**](javascript:__doPostBack('ctl00$ctl00$MainContentArea$MainContentArea$historyControl$ReorderHistoryLink','')) | | **Search Terms** |
| --- | --- | --- |
|  | S31 | S11 AND S30 |
|  | S30 | S12 OR S13 OR S14 OR S15 OR S16 OR S17 |
|  | S17 | hospital mortality |
|  | S16 | cause of death |
|  | S15 | (MH "Cause of Death") OR (MH "Hospital Mortality") |
|  | S14 | hospital admissions or hospitalization or hospitalisation or hospital stay or readmissions |
|  | S13 | hospital admission trends |
|  | S12 | (MH "Patient Admission") |
|  | S11 | S1 OR S10 |
|  | S10 | S4 AND S9 |
|  | S9 | S5 OR S6 OR S7 OR S8 |
|  | S8 | structured education |
|  | S7 | self management education or education or diabetes self management education program |
|  | S6 | patient education |
|  | S5 | (MH "Diabetes Education") OR (MH "Patient Education") |
|  | S4 | S2 OR S3 |
|  | S3 | diabetes mellitus type 2 or diabetes type 2 or t2dm or type 2 diabetic or t2d or diabetes 2¨ |
|  | S2 | (MH "Diabetes Mellitus, Type 2") |
|  | S1 | (MH "Diabetes Mellitus, Type 2/ED") |
