# Supplementary material for: Diabetes self‐management education and its association with hospital admissions and premature mortality: A scoping review and meta‐analysis
Source: Diabetes Obes Metab. 2025 Nov 24;28(2):850–64. doi: 10.1111/dom.70296 (PMC12803649; doi:10.1111/dom.70296)
Supplement: Supplementary file 1 — DATA S1. Supporting Information. [file DOM-28-850-s001.zip › Scoping review 2 PROTOCOL. V0.3 3.11.25.docx]

**Hospital admission and mortality outcomes in diabetes self-management education: a scoping review protocol.**

# Abstract

**Introduction**: Type 2 diabetes is associated with an increase in all-cause hospital admissions and premature mortality. Structured diabetes self-management education (DSME) is internationally recommended for individuals with type 2 diabetes and is associated with improved self-management skills, increased well-being, and short-term improvements in glycated haemoglobin (HbA1c). However, DSME’s impact on reducing hospital admissions and premature mortality is unclear. This scoping review aims to map the extent of literature exploring the relationship between DSME attendance, hospital admissions and mortality in adults with type 2 diabetes.

**Methods and analysis**: This review followed the PRISMA- ScR guidelines. Searches were conducted across three electronic databases on January 10, 2024, and updated September 2025: CINAHL, MEDLINE and EMBASE using core search terms for type 2 diabetes were combined using Boolean operators with terms relevant to diabetes self-management education, hospital admission and mortality. No restrictions on study design, quality, location, time, or sex. Reference lists of final documents will be hand searched for additional relevant literature. All articles will be assessed blind independently by two reviewers prior to data extraction and charted using a bespoke template.

**Ethics and dissemination**: Ethical approval is not required for this scoping review. Academic and clinical colleagues will be consulted to provide insight and feedback on study findings. Dissemination will include peer review publications in scientific journals and presentation at professional conferences.

# Introduction

Diabetes is a complex condition leading to disability and premature mortality with 7000 excess deaths in the United Kingdom in 2022^1^. Globally the number of adults living with diabetes has risen sharply over the past 40 years^2^ with the International Diabetes Federation estimating 537 million people are affected worldwide^3^ of whom approximately 4.3 million reside in the UK^4^. Over 90% have type 2 diabetes, with an increased prevalence and multimorbidity risk in areas with high deprivation, poor healthcare access, poorer housing, reduced finances, and lower educational attainment^2,4^. Individuals with type 2 diabetes are being diagnosed younger and living longer^2^, likely due to wide implementation of diabetes secondary prevention guidelines^5–8^, yet there remains a persistent gap in excess mortality compared to populations without the disease. Diabetes Self-Management Education (DSME) is internationally recognised as the cornerstone to successful diabetes self-management, and with pharmacotherapy, fundamental in achieving early glycaemic control and prevention of diabetes-related micro- and macrovascular complications^7,9^.

Landmark studies demonstrate the effectiveness of DSME in improving self-management skills, reducing diabetes distress and improving health related quality of life, with short to medium term improvements to glycated haemoglobin (HbA1c)^10–14^. Despite this, there remains limited and conflicting evidence on the longer-term benefits of DSME on disease progression. People living with type 2 diabetes frequently present with at least 1 comorbid condition which impacts ability to self-care^15^, disease progression, likelihood of hospital admissions and premature mortality^16^. Whilst a decline in diabetes related morbidity and mortality is evidenced within the literature^2,17,18^, twenty percent of all UK hospital beds are currently occupied by people with diabetes, a trend replicated globally with hospitalisation rates in those with diabetes 2-6 times higher than other populations^19,20^. Hospital bed usage is expected to rise to 25% by 2030^21^, most (92%) are admitted for conditions and illnesses other than diabetes^18,22^. Similarly, a diversification of causes of death and mortality rates have been identified globally, yet an unexplained excess mortality risk remains for individuals with diabetes^2,5,6,17^.

With increasing evidence suggesting primary and secondary management of diabetes is not enough to remove the persistent excess gap in hospital admissions and premature mortality, a comprehensive overview of the long-term impact of DSME, fundamental to successful self-management^9^, is warranted to understand the association with these critical outcomes. Therefore, the objective of this scoping review was to systematically map the available evidence, key concepts and existing knowledge on DSME, hospital admission and premature mortality in adults living with type 2 diabetes.

# Methods

The protocol for this scoping review was informed by the five-stage process for scoping studies methodological framework^23^ and developed using the Preferred Reporting Items for Systematic Reviews and Meta-Analysis Extension for Scoping Reviews^24^ (PRISMA-ScR) guidelines. Our overall review questions were:

- What is the association between DSME attendance and hospital admissions in people living with type 2 diabetes?
- What is the association between DSME attendance and premature mortality in people living with type 2 diabetes?

Following a population, intervention, comparator, outcome (PICO) framework to conceptualise the review focus as recommended in the PRISMA-ScR guidelines^23^ our population was adults living with type 2 diabetes, intervention was structured DSME, comparator was attendance at DSME versus no DSME or part completion, and outcome was hospital admissions or mortality.

## Eligibility criteria

This review will include studies involving adults aged 18 years or older with a confirmed diagnosis of type 2 diabetes. Studies focusing on diabetes prevention education will be excluded. All study designs examining DSME globally will be considered, provided that outcomes specific to type 2 diabetes are reported independently from other diabetes types or conditions. It is however recognised that prevalence, risks and approaches to supported self-management may differ widely outside of western countries.

No restrictions will be applied based on gender, sex, or ethnicity. Only studies published from 2004 onwards will be included. This cutoff reflects significant developments in DSME during this period. Although the National Institute for Health and Care Excellence (NICE) formally recommended structured DSME programs in 2006, relevant research and program implementations began emerging slightly earlier. Limiting the review to studies from 2004 ensures inclusion of contemporary evidence that reflects current educational standards and practices while maintaining the feasibility of the review. Studies must be written in English or have an English transcription available. When multiple publications report findings from the same study cohort, the most recent publication (e.g., a 5-year follow-up instead of a 2-year follow-up) will be selected.

DSME refers to behavioural interventions designed to support patients’ self-management skills in type 2 diabetes. However, no nationally or internationally standardised content exists. For this scoping review, a program will be classified as DSME if it meets the following criteria:

- It is evidence-based and structured, such as having an approved curriculum with clearly stated learning outcomes and objectives.
- It is delivered by trained educators.
- It is fundamentally distinct from routine patient advice typically provided during standard clinical consultations.

Accreditation of programs is not required for inclusion. Studies will be excluded if they focus solely on carbohydrate counting, pertain to type 1 diabetes, or primarily compare different methods of delivering education rather than evaluating the outcomes of DSME itself.

## Information sources and searches

To identify relevant evidence, the following bibliographic databases were searched: CINAHL, MEDLINE, and EMBASE. Search strategies were drafted and refined with the assistance of an experienced librarian prior to being conducted on 1 January 2024. Both primary studies and grey literature relevant to DSME for type 2 diabetes were targeted.

PubMed was not searched separately because its primary content substantially overlaps with MEDLINE, which provides indexed coverage of biomedical literature; therefore, additional yield from PubMed-only records was expected to be minimal. Web of Science was not included because the review focused on clinical and health sciences literature comprehensively covered by CINAHL, MEDLINE, and EMBASE. These databases were selected to ensure broad coverage of nursing, biomedical, and allied health research while avoiding redundancy across multidisciplinary citation databases.

Systematic reviews will initially be included to ensure that no potentially eligible studies are missed. All references will be exported into Rayyan software^25^ for screening and duplicate removal. Reference lists of included studies will be hand-searched to identify further relevant literature.

## Selection of sources of evidence

Before screening titles and abstracts, and to ensure consistency in applying the inclusion and exclusion criteria, two reviewers independently screened a random sample of 20 articles to assess inter-rater reliability using Cohen’s Kappa coefficient. Screening will proceed only once a Kappa score of ≥0.81 (indicating very good agreement) is achieved.

The full text of selected citations will be assessed in detail against the inclusion criteria by two independent reviewers. Reasons for exclusion of sources of evidence at full text that do not meet the inclusion criteria will be recorded and reported in the scoping review. Any disagreements that arise between the reviewers at each stage of the selection process will be resolved through discussion. Where agreement cannot be made a third reviewer will be consulted to resolve discrepancies. The results of the search and the study inclusion process will be reported in full in the final scoping review and presented in a PRISMA flow diagram

## Data extraction

A data extraction table will be developed, piloted, and refined as necessary following discussion between the two reviewers, using a random sample of 10 studies prior to full data extraction. Extracted data will include details on study participants, concept, context, methodology, and key findings relevant to the review questions. Table 1 outlines the preliminary data extraction plan.

Table 1: Preliminary data extraction plan

| Article information | Author, year, study location, type of publication |
| --- | --- |
| Study design | Aims and objectives, sample and sample size, methods |
| DSME characteristics | Details of facilitators, programme name, number of sessions, overall length, accreditations |
| Outcomes | Primary and secondary outcomes, post hoc assessments, dropout |
| Admission / mortality outcomes | Measurement, positive or negative impact of programme on outcome of interest. |
| Study limitations | Limitations in study design and data analysis |
| Conclusions and recommendations | Overall study conclusions and recommendations. Related studies. |

Studies will be grouped by outcome of interest and summarised according to setting, study design, outcome measures, hospital admissions, mortality data, and author conclusions. This approach will enable a comprehensive overview of the literature and highlight patterns or gaps related to DSME, hospital admissions, and mortality in adults with type 2 diabetes.

## Critical appraisal of individual sources of evidence

The Mixed Methods Appraisal Tool (MMAT) will be used to assess the methodological quality of included studies, providing a consistent framework suitable for this scoping review. Following MMAT guidelines, two reviewers will independently evaluate each paper, beginning with the screening questions and then proceeding to the relevant domain-specific criteria for randomised controlled trials (RCTs), non-randomised studies, or quantitative descriptive studies^26.^.

Rather than assigning an overall quality score, the appraisal will report detailed ratings (yes, no, or cannot tell) for each criterion to offer a detailed understanding of study quality. Studies will not be excluded based on methodological quality. Table 2 presents the preliminary plan for quality appraisal of included studies.

Table 2: MMAT appraisal plan

| Study type | Randomised controlled trial, quantitative non-randomised, quantitative descriptive | | | |
| --- | --- | --- | --- | --- |
| Screening questions (all studies) | Are there clear research questions?  Do the collected data allow the research question to be addressed? | Yes | No | Cannot tell |
| Randomised controlled trial questions | Is randomisation appropriately performed?  Are groups comparable at baseline?  Is outcome data complete?  Are outcome assessors blinded to intervention?  Did participants adhere to intervention? | Yes | No | Cannot tell |
| Quantitative non-randomised questions | Are participants representative of the target population?  Are measurements appropriate regarding outcome and intervention?  Are the outcome data complete?  Are the confounders accounted for in the design and analysis?  During the study is the intervention administered as intended? | Yes | No | Cannot tell |
| Quantitative descriptive questions | Is the sampling strategy relevant to address the research question?  Is the sample representative of the target population?  Are the measurements appropriate?  Is the risk of non-response boas low?  Is the statistical analysis appropriate to answer the research question? | Yes | No | Cannot tell |

## Data analysis and presentation

This scoping review is designed to provide a comprehensive overview of the extent and nature of literature examining the association between DSME attendance, hospital admissions, and premature mortality in adults with type 2 diabetes. Whilst the specific reporting will be organised and their structure determined by the results of the scoping review, the reporting and presentation of results is likely to include the following strategies:

- A modified PRISMA-ScR flowchart and checklist: This approach will ensure systematic and transparent documentation of the search process, study selection, data extraction, and synthesis, enhancing reproducibility and clarity for readers.
- Numerical Summary: A quantitative overview will be presented, detailing the number of studies included, categorised by study design (e.g., RCTs, quantitative descriptive studies), geographic location, publication year, and other relevant descriptors. This will provide a clear picture of the distribution and volume of evidence, highlighting trends over time and across regions or healthcare settings. Heterogeneity of DSME across studies will be discussed.
- Thematic Analysis and Visual Representation: Key themes emerging from the synthesised data will be presented as they relate to the scoping review key questions and objectives.

Together, these strategies will provide a rich, multi-dimensional understanding of the existing literature, inform future research priorities, and guide clinical practice and policy decisions related to DSME’s impact on hospital admissions and premature mortality in type 2 diabetes.

A narrative summary will accompany the tabulated and/or charted results and will describe how the results relate to the reviews objective and question/s.

## Funding

This scoping review received no specific grant from any funding agency in the public, commercial or not-for-profit sectors.

## Author contributions

## Conflicts of interest

No conflicts of interest are reported.

# References

1. Iacobucci G. Diabetes: Missed routine checks are causing premature deaths in England, charity warns. *BMJ*. 2023;381:1070. doi:10.1136/BMJ.P1070

2. Pearson-Stuttard J, Buckley J, Cicek M, Gregg EW. The Changing Nature of Mortality and Morbidity in Patients with Diabetes. *Endocrinol Metab Clin North Am*. 2021;50(3):357-368. doi:10.1016/j.ecl.2021.05.001

3. Federation ID. IDF Diabetes Atlas Tenth edition 2021. International Diabetes Federation. 2021. Accessed August 2, 2023. https://diabetesatlas.org/data/en/country/209/gb.html

4. Diabetes UK. *Diabetes Care: Is It Fair Enough?*; 2023. Accessed August 2, 2023. https://diabetes-resources-production.s3.eu-west-1.amazonaws.com/resources-s3/public/2023-05/DUK_Diabetes%20is%20Serious%20Report%202023.pdf

5. Amini M, Zayeri F, Salehi M. Trend analysis of cardiovascular disease mortality, incidence, and mortality-to-incidence ratio: results from global burden of disease study 2017. *BMC Public Health*. 2021;21(1):1-12. doi:10.1186/S12889-021-10429-0/TABLES/3

6. Laurberg T, Graversen SB, Sandbæk A, Wild SH, Vos RC, Støvring H. Trends in cause-specific mortality among people with type 2 and type 1 diabetes from 2002 to 2019: a Danish population-based study. *The Lancet Regional Health - Europe*. 2024;41. doi:10.1016/j.lanepe.2024.100909

7. Recommendations | Type 2 diabetes in adults: management  | Guidance | NICE.

8. QOF indicators | Goals and outcome measures | Diabetes - type 2 | CKS | NICE. Accessed August 3, 2023. https://cks.nice.org.uk/topics/diabetes-type-2/goals-outcome-measures/qof-indicators/

9. Davies MJ, Aroda VR, Collins BS, et al. Management of Hyperglycemia in Type 2 Diabetes, 2022. A Consensus Report by the American Diabetes Association (ADA) and the European Association for the Study of Diabetes (EASD). *Diabetes Care*. 2022;45(11). doi:10.2337/dci22-0034

10. Wong CKH, Wong WCW, Wan EYF, Wong WHT, Chan FWK, Lam CLK. Increased number of structured diabetes education attendance was not associated with the improvement in patient-reported health-related quality of life: Results from Patient Empowerment Programme (PEP). *Health Qual Life Outcomes*. 2015;13(1). doi:10.1186/s12955-015-0324-3

11. Wichit N, Mnatzaganian G, Courtney M, Schulz P, Johnson M. Randomized controlled trial of a family-oriented self-management program to improve self-efficacy, glycemic control and quality of life among Thai individuals with Type 2 diabetes. *Diabetes Res Clin Pract*. 2017;123. doi:10.1016/j.diabres.2016.11.013

12. Bukhsh A, Khan TM, Phisalprapa P, et al. Impact of Pharmacist-Led Diabetes Self-Care Education on Patients With Type 2 Diabetes in Pakistan: A Randomized Controlled Trial. *Front Pharmacol*. 2022;13. doi:10.3389/fphar.2022.754999

13. Khunti K, Gray LJ, Skinner T, et al. Effectiveness of a diabetes education and self management programme (DESMOND) for people with newly diagnosed type 2 diabetes mellitus: Three year follow-up of a cluster randomised controlled trial in primary care. *BMJ (Online)*. 2012;344(7860). doi:10.1136/bmj.e2333

14. Davies MJ, Heller S, Skinner TC, et al. Effectiveness of the diabetes education and self management for ongoing and newly diagnosed (DESMOND) programme for people with newly diagnosed type 2 diabetes: Cluster randomised controlled trial. *BMJ*. 2008;336(7642). doi:10.1136/bmj.39474.922025.BE

15. Nowakowska M, Zghebi SS, Ashcroft DM, et al. The comorbidity burden of type 2 diabetes mellitus: patterns, clusters and predictions from a large English primary care cohort. *BMC Med*. 2019;17(1). doi:10.1186/s12916-019-1373-y

16. JBDS. *Admissions Avoidance and Diabetes: Guidance for Clinical Commissioning Groups and Clinical Teams Produced by the Joint British Diabetes Societies for Inpatient Care (JBDS-IP)*.; 2013. http://www.diabetes.org.uk/Documents/Position%20statements/best-practice-commissioning-diabetes-services-integrated-framework-

17. Pearson-Stuttard J, Bennett J, Cheng YJ, et al. Trends in predominant causes of death in individuals with and without diabetes in England from 2001 to 2018: an epidemiological analysis of linked primary care records. *Lancet Diabetes Endocrinol*. 2021;9(3). doi:10.1016/S2213-8587(20)30431-9

18. Pearson-Stuttard J, Cheng YJ, Bennett J, et al. Trends in leading causes of hospitalisation of adults with diabetes in England from 2003 to 2018: an epidemiological analysis of linked primary care records. *Lancet Diabetes Endocrinol*. 2022;10(1). doi:10.1016/S2213-8587(21)00288-6

19. Comino EJ, Harris MF, Islam MDF, et al. Impact of diabetes on hospital admission and length of stay among a general population aged 45 year or more: A record linkage study. *BMC Health Serv Res*. 2015;15(1). doi:10.1186/S12913-014-0666-2,

20. AbuHammad GAR, Naser AY, Hassouneh LKM. Diabetes mellitus-related hospital admissions and prescriptions of antidiabetic agents in England and Wales: an ecological study. *BMC Endocr Disord*. 2023;23(1):1-16. doi:10.1186/S12902-023-01352-Z/TABLES/3

21. Diabetes UK. Making hospitals safe for people with diabetes. *Diabetic Medicine*. 2019;36(Supplement 1).

22. Diabetes Workstream - Getting It Right First Time - GIRFT. Accessed August 19, 2024. https://gettingitrightfirsttime.co.uk/medical_specialties/diabetes-workstream/

23. Arksey H, O’Malley L. Scoping studies: Towards a methodological framework. *International Journal of Social Research Methodology: Theory and Practice*. 2005;8(1):19-32. doi:10.1080/1364557032000119616

24. Tricco AC, Lillie E, Zarin W, et al. PRISMA extension for scoping reviews (PRISMA-ScR): Checklist and explanation. *Ann Intern Med*. 2018;169(7). doi:10.7326/M18-0850

25. Ouzzani M, Hammady H, Fedorowicz Z, Elmagarmid A. Rayyan-a web and mobile app for systematic reviews. *Syst Rev*. 2016;5(1). doi:10.1186/S13643-016-0384-4

26. Nha Q, Pluye P, Fàbregues S, et al. MIXED METHODS APPRAISAL TOOL ( MMAT ) VERSION 2018 User guide. Published online 2018.

## Appendix 1: CINAHL PLUS search strategy

| [**Search ID#**](javascript:__doPostBack('ctl00$ctl00$MainContentArea$MainContentArea$historyControl$ReorderHistoryLink','')) | | **Search Terms** | |
| --- | --- | --- | --- |
| S19 | S11 AND S18 | |  |
| S18 | S12 OR S13 OR S14 OR S15 OR S16 OR S17 | |  |
| S17 | hospital mortality | |  |
| S16 | cause of death | |  |
| S15 | (MH "Cause of Death") OR (MH "Hospital Mortality") | |  |
| S14 | hospital admissions or hospitalization or hospitalisation or hospital stay or readmissions | |  |
| S13 | hospital admission trends | |  |
| S12 | (MH "Patient Admission") | |  |
| S11 | S1 OR S10 | |  |
| S10 | S4 AND S9 | |  |
| S9 | S5 OR S6 OR S7 OR S8 | |  |
| S8 | structured education | |  |
| S7 | self management education or education or diabetes self management education program | |  |
| S6 | patient education | |  |
| S5 | (MH "Diabetes Education") OR (MH "Patient Education") | |  |
| S4 | S2 OR S3 | |  |
| S3 | diabetes mellitus type 2 or diabetes type 2 or t2dm or type 2 diabetic or t2d or diabetes 2¨ | |  |
| S2 | (MH "Diabetes Mellitus, Type 2") | |  |
| S1 | (MH "Diabetes Mellitus, Type 2/ED") | |  |

MEDLINE search strategy

| [**Search ID#**](javascript:__doPostBack('ctl00$ctl00$MainContentArea$MainContentArea$historyControl$ReorderHistoryLink','')) | | **Search Terms** |
| --- | --- | --- |
|  | S31 | S11 AND S30 |
|  | S30 | S12 OR S13 OR S14 OR S15 OR S16 OR S17 OR S18 OR S19 OR S20 OR S21 OR S22 OR S23 |
|  | S23 | social determinants of health |
|  | S22 | indicies of multiple deprivation |
|  | S21 | indicies of multiple deprivation |
|  | S20 | health deprivation |
|  | S19 | (MH "Health Inequities") OR (MH "Social Determinants of Health") |
|  | S18 | (MH "Social Deprivation") |
|  | S17 | hospital mortality |
|  | S16 | cause of death |
|  | S15 | (MH "Cause of Death") OR (MH "Hospital Mortality") |
|  | S14 | hospital admissions or hospitalization or hospitalisation or hospital stay or readmissions |
|  | S13 | hospital admission trends |
|  | S12 | (MH "Patient Admission") |
|  | S11 | S1 OR S10 |
|  | S10 | S4 AND S9 |
|  | S9 | S5 OR S6 OR S7 OR S8 |
|  | S8 | structured education |
|  | S7 | self management education or education or diabetes self management education program |
|  | S6 | patient education |
|  | S5 | (MH "Diabetes Education") OR (MH "Patient Education") |
|  | S4 | S2 OR S3 |
|  | S3 | diabetes mellitus type 2 or diabetes type 2 or t2dm or type 2 diabetic or t2d or diabetes 2¨ |
|  | S2 | (MH "Diabetes Mellitus, Type 2") |
|  | S1 | (MH "Diabetes Mellitus, Type 2/ED") |
