# Supplementary material for: Diabetes self‐management education and its association with hospital admissions and premature mortality: A scoping review and meta‐analysis
Source: Diabetes Obes Metab. 2025 Nov 24;28(2):850–64. doi: 10.1111/dom.70296 (PMC12803649; doi:10.1111/dom.70296)
Supplement: Supplementary file 1 — DATA S1. Supporting Information. [file DOM-28-850-s001.zip › Table S1 Extracted data for mortality.docx]

**Table S1: Extracted data for mortality**

| **Author & year** | **Country** | **Aims** | **Sample description** | **Outcome measures** | **Mortality** | **Author conclusions/ recommendations** | **DSME characteristics** |
| --- | --- | --- | --- | --- | --- | --- | --- |
| Adolfsson^28^, 2006 | Sweden | Impact of empowerment DSME on diabetes knowledge, self-efficacy and satisfaction. | 88 adults  Mean age 63.0  40.9% female | Diabetes knowledge, self-efficacy, satisfaction with daily life, BMI and glycaemic control | Deaths reported as drop out (intervention group 1/42, control group 1/46) | Empowerment approach education improved diabetes knowledge confidence and maintained glycaemic control at 1 year follow up in patients with ‘relatively adequate’ control at baseline. | Empowerment approach diabetes education  Face to face, group format  Delivered by physicians and diabetes nurse specialists  4-5 sessions totalling 10-12.5 hours    Patients must have completed a minimum of 60% to be included in analysis. 69% attended all sessions, 31% missed 1 session. |
| Blackberry^38^, 2013 | Australia | DSME effectiveness on glycaemic control | 473 adults  Mean age 62.8  43% female | HbA1c | Deaths reported as drop out ( intervention group 4/236, control group 4/237) | Telephone coaching education for patients with poorly controlled type 2 diabetes was no more effective than standard care on HbA1c. Higher proportion of patients in intervention group attained a healthy weight. | Patient engagement and coaching for health programme  Telephone and 1 face to face, 1:1 format  Delivered by practice nurses  9 sessions, total programme length not stated  Patients must have completed a minimum of analysis. ITT analysis used. Median 3 session attended. Only 6% attended 8 or more. |
| Chao^54^, 2014 | Taiwan | Does DSME improve self-care and medical outcomes | 520 adults  Mean age not stated  52.2% female | HbA1c and positive markers for diabetes complication rates | Deaths reported as drop out (intervention group 0/260, control group 7/260) | Educational programme significantly improved HbA1c levels however there was not a similar reduction in complication rates (serum creatinine, urine analysis, microalbumin, triglycerides, BP, cataracts or retinopathy) at 6 months. | Programme name not stated (Bespoke programme for elderly taiwanese population)  Face to face, group format  Delivered by nurses  3 sessions totalling 3 hours  Measurement of completion not stated. ITT analysis used |
| Clancy^32^, 2007 | USA | Effectiveness of group DSME on clinical outcomes | 186 adults  Mean age 56.1  72% female | HbA1c, lipid profiles and BP | Deaths reported as drop out ( intervention group 2/96, control group 1/90) | Group education sessions improved ADA key process of care adherence but no change in clinical outcomes. | Programme name not stated (developed from published curriculum)  Face to face, group format  Delivery directed by a physician  12 sessions totalling 24 hours    Measurement of completion not stated |
| Clark^55^, 2004 | UK | Develop, implement and evaluate DSME aimed at improving adherence to lifestyle recommendations | 100 adults  Mean age 59.5  42% female | Self-care skills, fat related dietary habits, physical activity, weight | Deaths reported as drop out ( intervention group 1/50, control group 2/50) | Brief tailored self-management intervention is effective on elements of dietary behaviour up to 12 months follow up. Self-care skills improved for both diet and physical health at 3 months; only physical health was maintained at 12 months. | Tailored self-management education  Face to face, group format  Facilitator background not stated  Number of sessions and programme length not stated  Measurement of completion not stated. ITT analysis used |
| Cooper^56^, 2008 | UK | Does DSME improve glucose control and effect change in self-care behaviours and illness beliefs. | 89 adults  Mean age 59  44% female | HbA1c, BMI, self-management skills | Deaths reported as drop out ( intervention group 2/53, control group 3/59) | DSME should be regarded as having broad patient-based outcomes and should not be expected to have lasting benefits on HbA1c. | Look after yourself empowerment programme  Face to face, group format  Delivered by nurses  8 sessions totalling 16 hours  Measurement of completion not stated. ITT analysis used. 15% attended less than 4 sessions |
| Crasto^40^, 2011 | UK | Does cardiovascular risk control & DSME benefit cardiovascular risk factors | 189 adults  Mean age 62.6  74% female | HbA1c, BP, lipids | Deaths reported as drop out ( intervention group 3/94, control group 5/95) | Interventions in glycaemic control should be underpinned by structured education promoting self-management in type 2 diabetes. | Programme name not stated (Based on DESMOND model)  Face to face, group format  Delivered by trained DESMOND educators  1 session totalling 3 hours  Measurement of completion not stated |
| Crowley^57^, 2013 | USA | Effect of CVD risk reduction in African American patients with type 2 diabetes | 359 adults  Mean age 56.5  72% female | HbA1c, SBP, LDL-C, medication adherence | Deaths reported as drop out ( intervention group 2/177, control group 2/182) | Education improved self-reported medication adherence but not cardiovascular risks in African Americans with type 2 diabetes. | Self-management education modules  Telephone, 1:1 format  Delivered by nurses  12 sessions totalling a mean 3 hours 40 (17.1 minutes per call)  Measurement of completion not stated. ITT analysis used. Mean 9.9 sessions completed. |
| Deakin^42^, 2006 | UK | Effectiveness of patient centred DSME on clinical, lifestyle and psychosocial outcomes. | 314 adults  Mean age 61.5  48% female | HbA1c | Deaths reported as drop out ( intervention group 3/157, control group 7/157) | X-PERT programme was shown at 14 months to improve HbA1c, Total cholesterol, body weight, BMI and waist size. | X-pert patient  Face to face, group format  Delivered by a diabetes dietician  6 sessions totalling 12 hours  Measurement of completion not stated. ITT analysis used. 81.5% attended 4 or more sessions |
| Edelman^58^, 2015 | USA | Effectiveness of DSME and hypertension management in community practices | 377 adults  Mean age 58.7  54.6% female | HbA1c and SBP | Deaths reported as drop out ( intervention group 4/193, control group 5/184) | Tailored nurse case management intervention for patients with diabetes and hypertension failed to improve SBP or HbA1c | Nurse behavioural management  Telephone, 1:1 format  Delivered by nurses  12 sessions, total number of hours not stated  Measure of completion not stated. 78% of sessions completed, mean 9.3. |
| Gagliardino^33^, 2013 | Argentina | Effectiveness of system intervention combined with physician and/or DSME on therapeutic indicators and costs in type 2 diabetes | 234 adults  Mean age 62  67% female | HbA1c | Deaths reported as drop out ( intervention group 31/117, control group 9/117) | Educational interventions in primary care can result in long term improvements to clinical, metabolic and psychological outcomes and is cost effective. | Programme name not stated  Face to face, group format  Delivered by trained educators  5 sessions totalling 6-9 hours  Measure of completion not stated. |
| Gamboa-Moreno^44^, 2019 | Spain | Efficacy of Spanish DSME versus usual care in T2DM in Spain | 157 adults  Mean age 64  40% female | HbA1c (cardiovascular risk factors; medication use; quality of life; self-efficacy; physical activity levels; GP appointments; hospitalisations) | Deaths reported as drop out ( intervention group 3/297, control group 4/297) | HbA1c reductions are difficult to obtain in adequately controlled patients. HbA1c as outcome may be more suitable for studying poorly controlled groups. | Spanish version, Chronic disease self-management programme (CDSMP)  Face to face format, not stated if group or 1:1  Delivered by healthcare professional and peer with lived experience of diabetes  6 sessions totalling 6 hours  80% attended a minimum of 4 sessions. ITT analysis used |
| Gehlawat^59^, 2019 | India | Effectiveness of DSME on self-care behaviours in type 2 diabetes | 314 adults  Mean age 54.9  64.4% female | Self-management skills | Deaths reported as drop out ( intervention group 0/157, control group 1/157) | Compliance with diabetes footcare and elements of dietary self-care significantly improved following educational intervention. | Programme name not stated (based on guidelines by the Indian Council of medical research, ADA & American Association of Diabetes Educators)  Face to face, group format  Delivered in part by a physician (for 2 x 45 min sessions)  8 sessions, total programme length not stated  Measure of completion not stated. ITT analysis used |
| Goudswaard^34^, 2004 | Netherlands | Short- and long-term efficacy of a 6-month DSME programme for T2DM. | 58 adults  Mean age 65.3  66% female | HbA1c | Deaths reported as drop out ( intervention group 1/28, control group 2/30) | Education effective in reducing HbA1c and delaying insulin therapy for patients on maximum oral therapy, however effect reduced at 1 year. Short term education requires regular reinforcements. | Programme name not stated (developed in collaboration with Dutch Foundation of Diabetes Nurses)  Face to face, 1:1 format  Delivered by diabetes nurse  6 sessions totalling 2.5 hours  Completion based on 100% attendance |
| Hamid^60^, 2015 | Samoa | Examine the impact of DSME on diabetes control and healthcare usage 1 year post intervention | 268 adults  Mean age 55  67% female | Primary care physician visits, emergency department attendance and hospitalisations | Deaths reported as drop out ( intervention group 2/164, control group 2/164) | DSME significantly increased primary care visits and decreased emergency department visits amongst those with high emergency department usage the previous year. | Programme name not stated (modelled on National Diabetes Education Program)  Face to face, 1:1 format  Delivered by a community health worker  Number of sessions and programme length not stated  Measure of completion not stated. ITT analysis used |
| Jaipakdee^35^, 2015 | Thailand | Using the RE-AIM framework to evaluate effectiveness of DSME | 403 adults  Mean age 61.3  76.7% female | HbA1c, health behaviour, depression and quality of life | Deaths reported as drop out ( intervention group 0/203, control group 2/200) | Significant improvements in glycaemic control, health behaviour, body weight and QOL were observed in intervention group suggesting DSME with computer assisted instruction is beneficial. | Diabetes self-management support education with computer assisted instruction programme    Face to face, group format  Delivered by nurses  6 sessions totalling 18 hours  Measure of completion not stated, ITT analysis used |
| Khunti^13^, 2012 | UK | Are benefits of a single DSME programme sustained at 3 years | 824 adults  Mean age 59  44.9% female | HbA1c, blood pressure, weight, blood lipid levels, smoking status, physical activity, quality of life, beliefs about illness, depression, emotional impact and drug use | Deaths reported as drop out ( intervention group 15/437, control group 11/387) | Despite sustained improvements in illness beliefs at three years, no difference in biomedical or lifestyle outcomes. | DESMOND  Face to face, group format  Delivered by healthcare professionals  1 session totalling 6 hours  Measurement of completion not stated |
| McGowan ^46^, 2015 | Canada | Effectiveness of peer led DSME on self-efficacy and behaviours | 252 adults  Mean age 64.2  61.8% female | HbA1c | Deaths reported as drop out ( intervention group 2/130, control group 4/122) | Fatigue, cognitive symptoms management, self-efficacy, communication with physicians and empowerment improved significantly. | Stanford Diabetes self-management programme  Face to face, group format  Facilitator background not stated  6 sessions totalling 12 hours  Measurement of completion not stated |
| Pearson^27^, 2021 | UK | Effectiveness of nurse led DSME on all-cause mortality | 160 adults  Mean age 74.5  34% female | Mortality | Over a course of 42.6 month follow up mortality in people with type 2 diabetes was reduced to 33% compared to 51% in those who did not receive DSME (p=0.025). Cardiovascular deaths, leading cause of mortality, was lower in intervention group (p<0.01). | DSME following ambulance call out for sever hypoglycaemia can significantly reduce cardiovascular mortality over a short to medium follow up period. | Programme name not stated  Face to face, group or 1:1 format not stated  Delivered by nurses  1 session, programme length not stated  Measurement of completion not stated |
| Perez-Escamilla^36^, 2015 | USA | Impact of DSME on glycaemic control in Latinos with type 2 diabetes | 211 adults  Mean age 56.3  73.5% female | HbA1c | Deaths reported as drop out ( intervention group 2/ 105 VS control group 1/106) | DIALBEST is an effective intervention to improve blood glucose in Latinos with T2DM. | DIALBEST (Diabetes among Latinos Best Practice Trial education)  Face to face, 1:1 format  Delivered by a community health worker  17 sessions, totally programme length not stated  Measurement of completion not stated |
| Perman^61^, 2011 | Argentina | Compare all-cause mortality in elderly patients living with type 2 diabetes compared to those who did not attend | 1730 adults  Mean age 73.0  48% female | All-cause mortality | All-cause mortality rate was 3.06 (2.39-3.91) for intervention group vs 5.53 (4.04-5.07) per 100 person/years for control group .  Crude hazard ratio for exposure to DSME was 0.68(0.52-0.88); P=0.004. Following adjustment for covariates this effect reduced from 33% to 18% (HR 0.82; 95% CI: 0.61-1.08) P=0.170. | Patients who attended DSME had a 33% lower all-cause crude mortality rate at 6 years of follow up. | Programme name not stated (modelled on chronic care model with additional diabetes content)  Face to face, group format  Delivered by a family physician or endocrinologist  4 sessions totalling 8 hours  Completion based on 75% attendance |
| Prezio^62^, 2013 | USA | Long term cost effectiveness of DSME by community workers in uninsured Mexican Americans. | 180 adults  Mean age 47  64.4% female | HbA1c, micro- and macrovascular complications, mortality | Mortality reduction simulated over a 20-year time horizon showed a minimal non-significant reduction in all-cause mortality – intervention group 20.41% (19.26, 22.03) control group 20.66(19.04, 21.78).  Similar outcomes reported for CHD mortality intervention group 3.74% (3.11, 4.37) control group 4.02(3.35, 4.69).  CVA mortality intervention group 1.11% (0.75, 1.46) vs control group 1.15(0.8, 1.5). | Simulated trial suggests a community health worker led diabetes education program is a cost-effective way to reduce diabetes complications (HbA1c, diabetic foot ulcers, foot amputations) over a 20-year horizon in uninsured Mexican Americans. | Community Diabetes Education Program (CoDE)  Face to face, 1:1 format  Delivered by a community health worker  7 sessions totalling 7 hours  Measurement of completion not stated |
| Rygg^63^, 2012 | Norway | Efficacy of an ongoing group DSME for type 2 diabetes | 146 adults  Mean age 66  45% female | HbA1c and patient activation | Deaths reported as drop out ( intervention group 1/73 VS control group 1/73) | DSME improved diabetes knowledge and some self-management skills, prevented an increase in HbA1c and can have a positive impact on patients with an increased HbA1c level. | Programme name not stated  Face to face, group format  Delivered by a nurse and dietician  3 sessions totalling 15 hours  Measurement of completion not stated, ITT analysis used |
| Samuel-Hodge^64^, 2009 | USA | Develop and test a church based DSME | 201 adults  Mean age 58.8  64% female | Dietary, physical activity, medication and glucose self-monitoring behaviours | Deaths reported as drop out ( intervention group 1/117 VS control group 1/84) | Diabetes knowledge and quality of life improved significantly in those attending DSME, and short-term significant reduction in HbA1c. | Programme name not stated  Face to face, group format  Delivered by a church diabetes advisor and peer supporter  12 sessions totalling 18-24 hours  Measurement of completion not stated, ITT analysis used. Mean 6.24 sessions completed |
| Sarkadi^65^, 2004 | Sweden | Effectiveness of DSME at 24 months and pinpoint mediators that may play a role in positive metabolic outcomes | 77 adults  Mean age 66.5  % female not reported | HbA1c | Deaths reported as drop out ( intervention group 1/39 VS control group 1/38) | DSME significantly decreased HbA1c 24 months after baseline and an increase in physical activity. Early intervention effects were followed by relapse at 12 months and a new significant decrease at 24 months. Follow up of educational initiatives should involve several consecutive measurements to capture possible late effects. | Programme name not stated  Face to face, group format  Delivered by a pharmacist and nurse  12 sessions, programme length not stated  Measurement of completion not stated , ITT analysis used |
| Sperl-Hillen^37^, 2013 | Spain | Are outcomes sustained in patients with suboptimal control | 623 adults  Mean age 62  49% female | HbA1c | Deaths reported as drop out ( intervention group 6/489 VS control group 2/134) | Individual education resulted in long standing improvement to self-efficacy and diabetes distress.  Group education improved patients’ diabetes understanding fin the short term however results were not sustained at 12 months.  There were no significant changes to HbA1c from either group or individual education when compared to control. | US Diabetes Conversations Maps  Face to face, group or 1:1 format  Delivered by a nurse or dietician  3 sessions totalling 3 hours  Measure of completion not stated. ITT analysis used. Of the 489 randomised to intervention 386 completed all the education, 62 part-completed and 41 did not attend |
| Trento^66^, 2004 | Italy | To study time course changes in knowledge, problem solving ability, and quality of life in patients attending DSME | 112 adults  Mean age 61.5  45.5% female | Diabetes knowledge, quality of life, HbA1c, BMI and HDL cholesterol | Deaths reported as drop out ( intervention group 3/56 vs 3/56) | Diabetes education groups improved their diabetes knowledge, problem solving ability at 2 years post intervention.  BMI decreased and HbA1c levels were maintained at 5-year follow up in intervention group (HbA1c levels increased in control group). | Programme name not stated  Face to face, group format  Delivered by a physician  11 sessions, programme length not stated    Measure of completion not stated |
| Wong^47^, 2015 | Hong Kong | Do patient empowerment programmes reduce all-cause mortality in type 2 diabetes | 27,278 adults  Mean age 63  57% female | First CVD event (CHD, stroke, heart failure, all-cause mortality) | Education participants had a lower rate of all-cause mortality (HR 0.564, 95% CI 0.445-0.715, P<0.001) than those who did not receive education.  All-cause mortality intervention group 113/13,639 vs 222/13,639). | Participating in DSME is associated with a decreased all-cause mortality and CVD events, especially stroke and heart failure in patients with type 2 diabetes over a 5 year follow up. Programme completion was related to reduction in CVD events. | Patient empowerment programme (PEP)  Face to face, group format  Delivered by nurse and community experts  6-8 sessions totalling 15 hours  Patients must have completed at least 1 session to be included in analysis, ITT analysis used |
| Wong^31^, 2016 | Hong Kong | Macrovascular and microvascular disease in obese patients with type 2 diabetes attending a patient empowerment programme | 6,372 adults  Mean age 65  58.5% female | All-cause mortality, first macrovascular event, first microvascular event, composite macro- and microvascular event | During a 31 month follow up PEP participants had a lower incidence rate of all-cause mortality (HR0.589, 95% CI 0.389-0.915, P= 0.018).  All-cause mortality intervention group 34/3186 vs control group 61/3186). | Enrolment to PEP was an effective approach to reduce all-cause mortality in type 2 diabetes | Patient empowerment programme (PEP)  Face to face, group format  Delivered by nurse and community experts  6-8 sessions totalling 15 hours  Patients must have completed at least 1 session to be included in analysis, ITT analysis used |

CVD: Cerebrovascular disease. CHD: Coronary heart disease. ITT: intention to treat.
