# Supplementary figures and images for: Diabetes self‐management education and its association with hospital admissions and premature mortality: A scoping review and meta‐analysis
Source: Diabetes Obes Metab. 2025 Nov 24;28(2):850–64. doi: 10.1111/dom.70296 (PMC12803649; doi:10.1111/dom.70296)

# Meta-analysis of Admission Rate Ratios

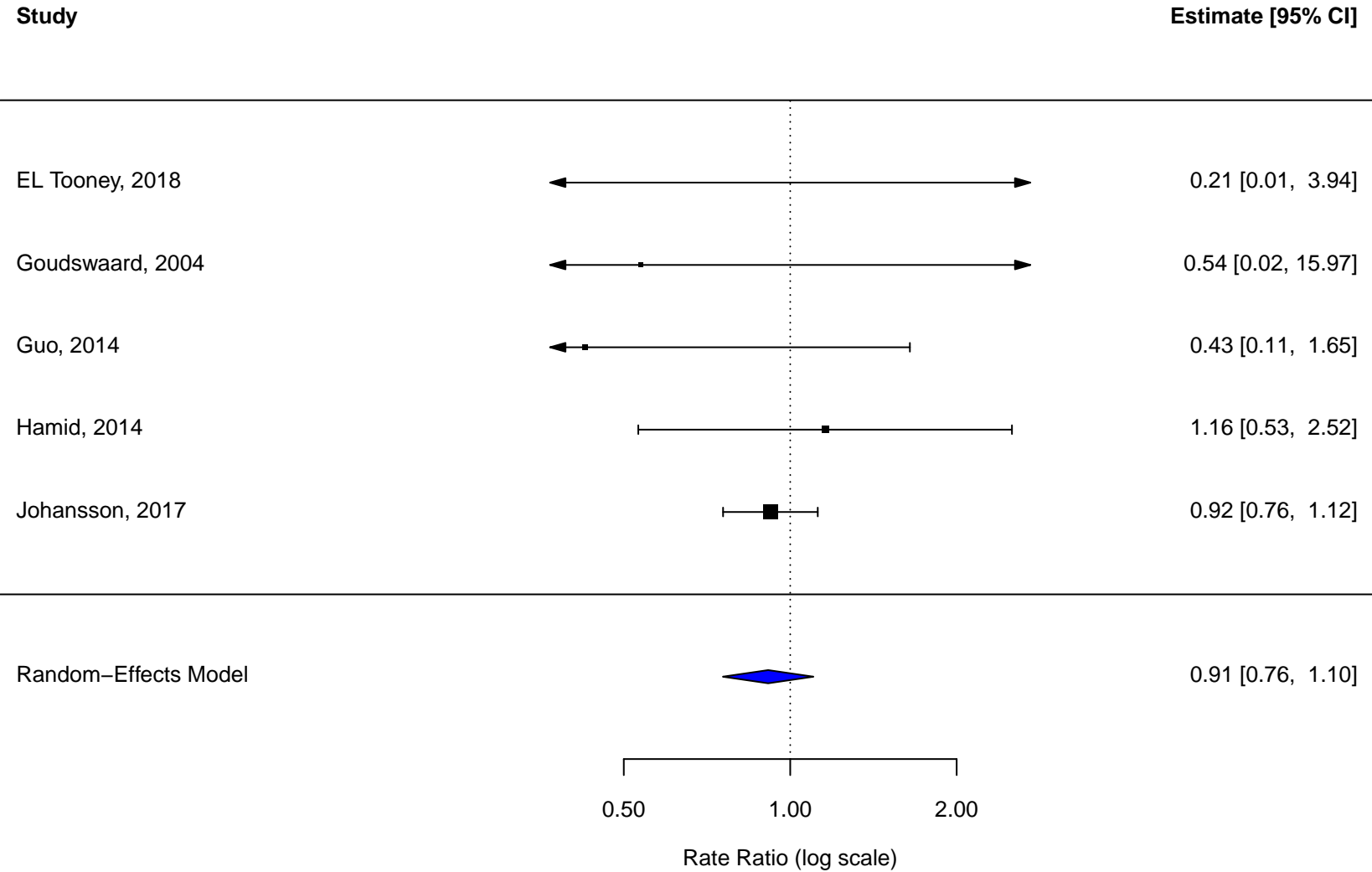

Supplement: Supplementary file 1 — DATA S1. Supporting Information. [file DOM-28-850-s001.zip › meta-analysis of admission rates based on count data.pdf]
